# Menstrual Health in Servicewomen: The Menstrual Cycle, Menstrual Disturbances, and Occupational Consequences

**DOI:** 10.1186/s40798-025-00963-1

**Published:** 2026-01-05

**Authors:** Ritva S. Mikkonen, Holly McClung, Gabrielle Giersch, Jeroen Van Cutsem, Helen Wright, Thomas J. O’Leary, Julie P. Greeves

**Affiliations:** 1 Faculty of Sport and Health Sciences, University of Jyväskylä, Sports Technology Unit Vuokatti, Vuokatti, Finland; 2 Military Performance Division, US Army Institute of Environmental Medicine, Natick, MA USA; 3 Thermal Mountain and Medicine Division US Army Institute of Environmental Medicine, Natick, MA USA; 4 VIPER Research Unit, Royal Military Academy, Brussels, Belgium; 5 Canadian Armed Forces Health Services, Canadian Armed Forces, Ottawa, Canada; 6 Army Health and Performance Research, Army Headquarters, Andover, SP11 8HT UK

**Keywords:** Hormones, Musculoskeletal injury, Nutrition, Estradiol, Performance

## Abstract

**Background:**

Most women serving in the military do so during their reproductive life and enter service at a young gynecological age. This review provides an overview of the menstrual cycle and summarizes the evidence for menstrual cycle disturbances in the military and how these disturbances to the menstrual cycle impact health and performance in the military.

**Main Text:**

Servicewomen often manage the practical challenges of menstruation and symptoms of the menstrual cycle or menstrual disturbances/dysfunction in an austere environment with no formalized support and/or education, and with unknown stigma and risks. Menstrual health in the military context implies that those who experience a menstrual cycle can access timely information, diagnosis, and support/treatment to achieve “*a state of complete physical*,* mental*,* and social well-being and not merely the absence of disease or infirmity*,* in relation to the menstrual cycle.”* Herein we describe how menstrual health is impacted in a multistressor environment, including nutrition (energy balance and energy availability, micronutrients, and microbiome), physical activity, and recovery (occupational tasks, sleep, psychological stress, environment), and how menstrual disturbances can affect occupational performance and the lived experience of the female workforce.

**Conclusions:**

We call for action of militaries worldwide to protect the health of Servicewomen to maximize their potential. Low representation, relatively recent full integration of women into the military workforce, and the exclusion of women from military research have led to policies developed from evidence on men, with the potential to impact the health and performance of Servicewomen.

## Key Points

1. Servicewomen face unique physiological challenges. Often, they must meet sex-neutral physical employment—or fitness—standards to be employed, and manage menstrual health.
2. A bidirectional relationship exists between multistressor military environments and menstrual health, whereby multistressors can influence endocrine function and menstrual health can be an additional stressor.
3. More high-quality research investigating the many aspects of menstrual health on the overall health and performance of Servicewomen is essential.

## Women in the Military and Menstrual Health

Women have been present on the battlefield for centuries and were formally allowed to join the Armed Forces, mainly in nursing roles, during the World Wars. Over the past decade, many countries (UK, US, Canada, Australia, Finland) have opened all roles to women, from fast-jet pilots, submariners, to ground close combat—roles that ‘close with and kill the enemy.’ Women, like men, can be trained to meet sex-neutral physical employment standards (depending on military role and country); they need to be physically strong, aerobically fit, and mentally resilient, whilst avoiding injury.

Although women comprise ~ 50% of the global population, they are the minority in the military; for example, women comprise approximately 12% of the total population in the UK Armed Forces [1]. The representation of women is lowest in ground close combat roles (< 3%) creating additional challenges in supporting women to maintain operational fitness. The low representation, recent full integration of women into the military workforce, and the exclusion of women from high quality military research [2, 3], have led to policies developed from evidence on men, with the potential to impact the health and performance of Servicewomen.

Menstrual health of Servicewomen [4] and overall effects of menstrual health, or lack thereof, on physical performance [5, 6] are not well researched. Menstrual health of Servicewomen has been identified as a priority research area [7] and many Servicewomen have been identified as desiring menstrual cycle suppression [8]. Most women enter service at a young gynecological age, relatively near (within ~ 5 years) *menarche* (first menstrual period), and living some or most of their reproductive life in the military; they often manage the practical challenges of menstruation and symptoms of the menstrual cycle in an austere environment with no formalized support and/or education, and with unknown stigma and risks. The aims of this paper are to provide an overview of the menstrual cycle and menstrual cycle disturbances, and review the evidence for the prevalence of menstrual disturbances in Servicewomen, the multistressors experienced by Servicewomen (including energy deficit, micronutrient deficiencies, microbiome, sleep, psychological stress, and environmental stress), and the impact of menstrual disturbances on musculoskeletal injury risk and physical and cognitive performance. Articles were sourced using MEDLINE and Google Scholar using key terms related to “Servicewomen” and “menstrual cycle”, “menstrual disturbances”, “hormones”, “energy balance”, “energy deficits”, “micronutrients”, “microbiome”, “sleep”, “psychological stress”, “heat”, and “cold”, and outcomes related to musculoskeletal injury, physical performance, and cognitive performance. Author expert knowledge of the area was also used to source relevant publications. Where there were no available data, other models, such as hormonal contraceptives, were explored to determine the effect of endocrine status on relevant outcomes. The paper concludes with a call for action of militaries worldwide to protect the health of Servicewomen to maximize their potential. The focus of this paper is on the menstrual cycle and menstrual disturbances, while acknowledging that menstrual disorders and hormonal contraceptive use require attention but go beyond the scope of the current paper. We use the terms “women” and/or “female” in line with the literature presented, which generally refers to birth sex. We also refer to “Servicewomen” in line with this terminology.

## Menstrual Health

Menstrual health is broadly defined as *“…a state of complete physical*,* mental*,* and social well-being and not merely the absence of disease or infirmity*,* in relation to the menstrual cycle.”* [9]. The military environment presents unique challenges for preserving menstrual health, and as such we have adapted Hennegan’s definition [9] in our efforts to a develop an evidenced framework to optimize the menstrual health of Servicewomen (Table 1).

**Table 1:** Definition of menstrual health for servicewomen (modified from [9], modifications in bolded italics)

Menstrual Health: ‘Menstrual health is a state of complete physical, mental, and social well-being and not merely the absence of disease or infirmity, in relation to the menstrual cycle.’ Achieving menstrual health in the military context implies that women and all other people who experience a menstrual cycle can:
• Access accurate, timely, age-appropriate information about the menstrual cycle, menstruation, and changes experienced throughout the life-course ***and specifically in the military environment***, as well as related self-care ***and hygiene practices in austere environments lacking privacy.***
• ***Access accurate and timely information about the influence of menstrual disturbances and dysfunction on operational tasks as well as the risks and benefits of menstrual suppression via hormonal contraceptives and family planning (hormonal and non-hormonal contraceptive methods).***
• Care for their bodies during menstruation such that their preferences, hygiene, comfort, privacy, and safety are supported ***with efficiency and discretion in austere environments.*** This includes accessing and using effective and affordable menstrual materials, ***transporting and storing materials in military environments***, and having supportive facilities and services, including water, sanitation, and hygiene services, for washing the body and hands, changing menstrual materials, and cleaning and / or disposing of used materials.
• Access timely diagnosis, treatment, and care for menstrual cycle-related discomforts and disorders ***(disturbances and dysfunction)***, including access to appropriate health services and resources, pain relief, and strategies for self-care ***in austere environments***.
• Experience a positive and respectful environment in relation to the menstrual cycle, free from stigma ***(cultural barriers)*** and psychological distress, including the resources and support they need to confidently care for their bodies ***(adequate sleep and nutrition)*** and make informed decisions about self-care throughout their menstrual cycle ***at all levels within the Armed Forces.***
• Decide whether and how to participate in all spheres of life, including civil, cultural, economic, social, and political, during all phases of the menstrual cycle, free from menstrual-related exclusion, restriction, discrimination, coercion, and / or violence. ***Understand the possible influence of the menstrual cycle and menstrual disturbances (as well as menstrual dysfunction and / or hormonal contraceptive use) on performance in essential military tasks.***

*We encourage readers to consider that transgender and gender-diverse individuals may also menstruate, experience menstrual disturbances, and / or have unique hormonal profiles that influence their health and performance. Furthermore, we recognize the challenges related to reaching populations for whom menstrual health is essential but who do not identify as female or a women

## The Menstrual Cycle

**Fig. 1:**
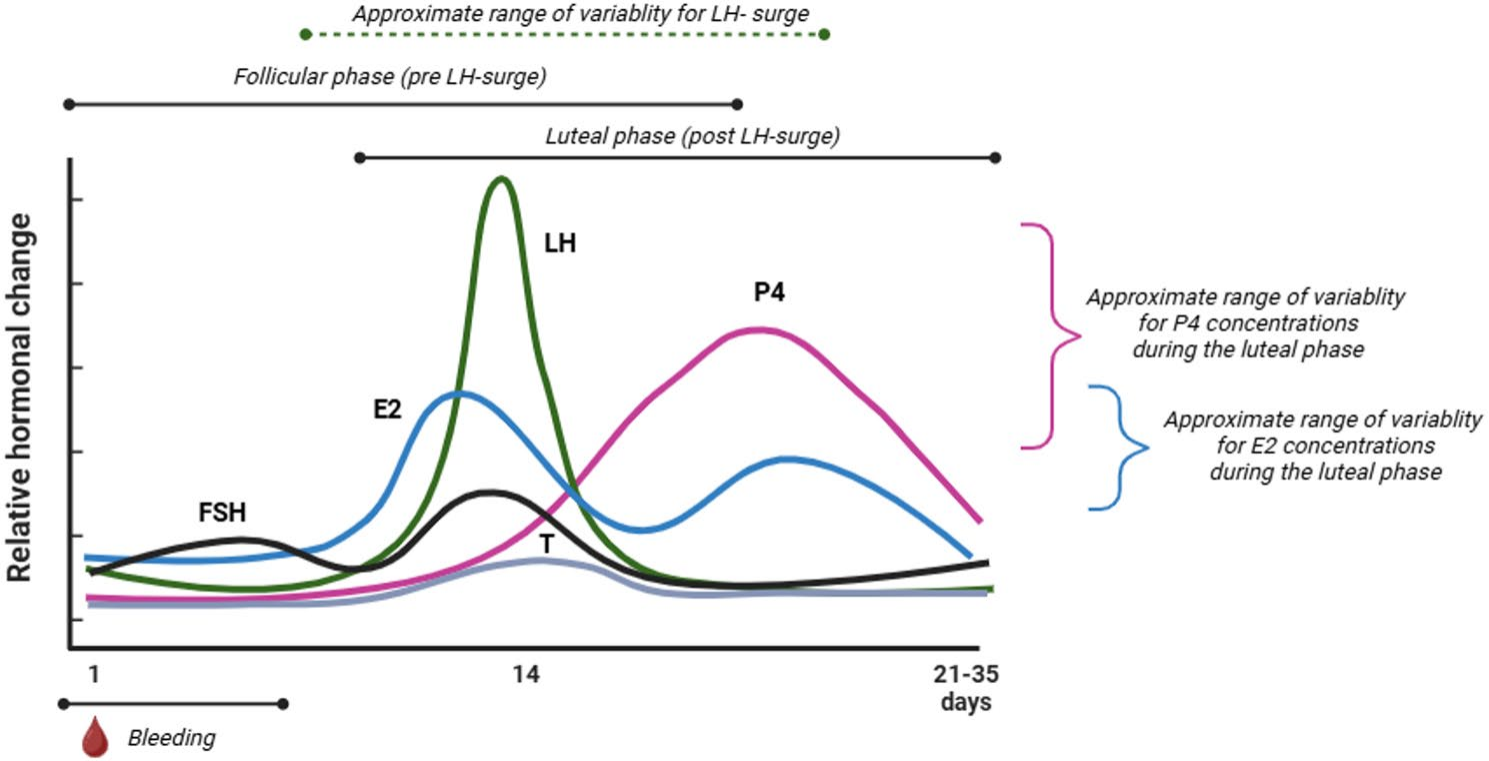
The ‘normal’ (eumenorrheic) menstrual cycle. *E2* 17β-estradiol, *FSH* follicle stimulating hormone, *LH* luteinizing hormone, *P4* progesterone, *T* testosterone. Figure created in Biorender

A “normal” or eumenorrheic menstrual cycle is generally between 21 and 35 days long and results in nine or more consecutive menstrual cycles per year. A eumenorrheic cycle (Fig. 1) occurs on a continuum, but features two basic phases: (1) the follicular phase, which commences with menstrual bleeding (menses), and is characterized by lower concentrations of progesterone (P4) and gradually increasing concentrations of 17β-estradiol (E2), and; (2) the luteal phase, which is characterized by higher concentrations of P4 and E2 that decrease prior to a new cycle in the absence of fertilization. These phases are often separated by a surge in luteinizing hormone (LH) that often triggers ovulation [10]; although, the timing of the LH surge [11] and the length of the luteinization process [12] may be variable. In the follicular phase, E2 stimulates growth of the follicle, which is primarily responsible for the proliferation and growth of the endometrial lining as well as vaginal lubrication. In the luteal phase, P4 prepares the endometrium for potential fertilization and pregnancy by maintaining endometrial stability and vascularization. Menstrual cycle phase lengths vary in regular / healthy menstrual cycles [13] where follicular phase length tends to be more variable than luteal phase length [14].

The menstrual cycle may be accompanied by physical and emotional symptoms that can influence daily wellbeing and performance. Transient adverse symptoms during the menstrual cycle that do not cause marked impairment, are generally considered “normal” [15], whereas symptoms such as those indicative of premenstrual syndrome (PMS) and premenstrual dysphoric disorder (PMDD) have standard clinical diagnostic criteria [15, 16] and may be problematic. Adverse symptoms are widespread both in the general population [17] and in military cadets [18]. During the early follicular phase (menses) these symptoms may include bleeding, cramping, and pain in the abdominal or pelvic region. Pain may radiate into the lower back and legs while bloating, sore breasts, headache, fatigue, and gastrointestinal symptoms may also be experienced. In the luteal phase, 7 to 10 days before the onset of menses, symptoms of PMS may manifest. Common physical symptoms of PMS include, but are not limited to, bloating, cramps or abdominal pain, and breast tenderness, while emotional symptoms may include irritability, mood swings, tension, depression, tearfulness, anxiety, angry outbursts, withdrawal from normal activities, and hypersomnia/insomnia [19, 20]. Symptoms from the early follicular or luteal phase are experienced by approximately 90% of women where PMS negatively impacts the daily life of 15–20% of women [19]. Between 3 and 8% of women experience more difficult psychological symptoms (PMDD) [21]. While premenstrual symptoms are associated with a decrease in P4 [21] and low-grade inflammation, as indicated by high-sensitivity C-reactive protein (10%) [22], the etiology of PMS/PMDD is not fully understood [23].

Estradiol and P4 have roles beyond reproduction. These hormones influence bone, muscle, nervous, epithelial, and connective tissues, as well as physiological processes such as metabolism, respiration, cardiovascular function, immunity, gastrointestinal and genitourinary function, neural function, and cognition [24–28]. Due to the pleiotropic effects of E2 and P4, it is reasonable to theorize that changes in reproductive function may have broad effects within the female body, including physical and cognitive performance, as well as musculoskeletal injury risk. Military trainees who report changes in menstrual cycles, indicate that menstrual and premenstrual symptoms impact academic, physical, and military activities [18]. Likewise, the menstrual cycle may have a small influence on physical performance [29] and perceived performance [30, 31], although the influence of the menstrual cycle on military-specific tactical, physical, and cognitive performance is not well understood. It is reasonable to postulate that individuals with a greater number of symptoms may experience transient adverse performance/wellness outcomes [32–34]. While the severity and nature of symptoms experienced during the menstrual cycle are highly individual, they may have implications for menstrual health and military performance. Even a “normal” menstrual cycle may have implications for military performance beyond logistical concerns related to menstrual hygiene.

### Menstrual Cycle Regulation

**Fig. 2:**
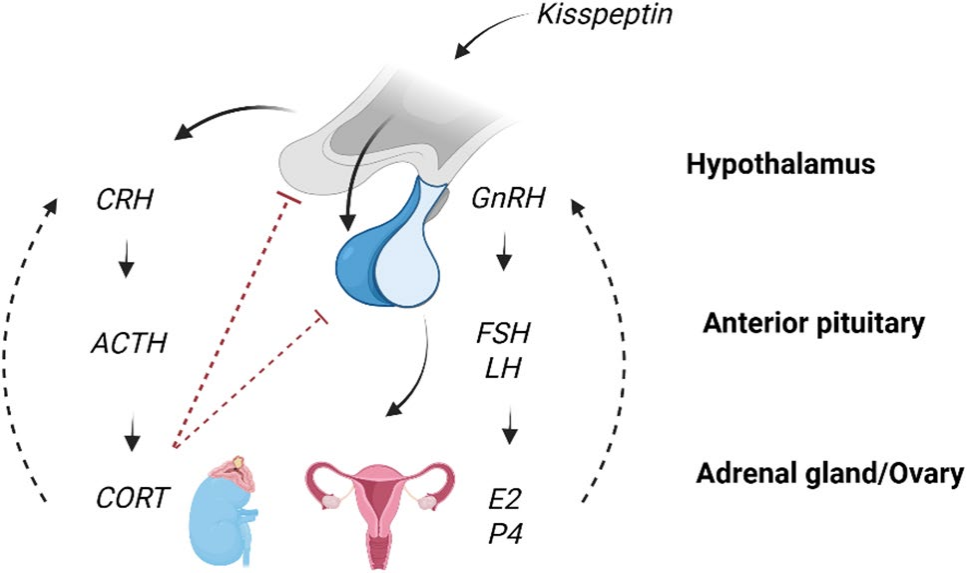
Endocrine regulation of the menstrual cycle. Hypothalamic-pituitary-ovarian axis and hypothalamic-pituitary-adrenal axis. *E2* 17β-estradiol, *FSH* follicle stimulating hormone, *GnRH* gonadotrophin releasing hormone, *LH* luteinizing hormone, *P4* progesterone, *CRH* corticotropin-releasing hormone. *ACTH* adrenocorticotropic hormone, *CORT* cortisol. Figure created in Biordender

The menstrual cycle is regulated centrally by the hypothalamic-pituitary-ovarian (HPO) axis, under influence of higher cortical control and negative feedback (Fig. 2). The kisspeptinergic system stimulates hypothalamic release of gonadotropin-releasing hormone (GnRH), which acts on the pituitary to stimulate release the gonadotropins: LH and follicle stimulating hormone (FSH). These gonadotropins stimulate the release of estrogens (estrone, E1 and estriol, E3 in addition to E2) and P4 from the ovaries. Estrogens, and specifically E2, are responsible for both primary and secondary female sex characteristics in addition to reproductive function [35]. The kisspeptinergic system, and thus the HPO-axis, is influenced by metabolic (leptin, ghrelin, insulin) and reproductive (estrogens, progesterone, testosterone) inputs as well as stress (the hypothalamic-pituitary-adrenal [HPA] axis; corticotropin releasing hormone [CRH] and cortisol) [36].

A bidirectional relationship exists between the HPA and HPO axes where estrogens modulate the response of the HPA axis and the HPA axis can inhibit estrogens secretion [37]. Elevated circulating cortisol produced by the HPA axis suppresses pituitary gonadotroph responsiveness to hypothalamic input, which may contribute to disruptions in HPO axis function [38–40]. Cortisol, a catabolic and glucoregulatory hormone, is secreted in response to physical stress and other challenges to body homeostasis [41–43] that may be encountered in military environments [44] where physical [45, 46], cognitive [47], and environmental stressors [48] may be combined. The HPA axis responds to both anticipated and actual demands/stressors where secretion of glucocorticoids mobilizes energy reserves to facilitate responses to these stressors [49] and the context of stress determines if the acute or chronic stress response results in positive or negative adaptations [49]. While stress response is determined by several factors such as genetics, life experience, environment, age, and sex [49], repeated and chronic stress are particularly detrimental to HPO-axis function [37].

### Menstrual Disturbances and Dysfunction

The tightly regulated and responsive HPO axis is susceptible to dysregulation from internal and environmental insults, leading to menstrual disturbances. The potential impact of menstrual disturbances (and dysfunction) on performance of Servicewomen warrants better understanding of their aetiology originating from exposure to military stressors. The ‘normal’ menstrual cycle has a rhythmic sequence of events, described above, that demonstrates adequate communication between the brain (hypothalamus and pituitary), ovary, and uterus [10, 50]. Secondary *menstrual disturbances*, also known as ovulatory disorders [51], are characterized by suppressed HPO function, or miscommunication between the brain, ovary and uterus, originating at the hypothalamus, that appears to occur concomitantly with disturbances in other hormonal axes [6] and which may result in abnormal uterine bleeding (AUB) [51]. Menstrual disturbances in a military setting may be related to intentional or unintentional low energy availability [52]—caused by a mismatch in energy intake and energy expenditure during arduous training—but may also be related to other occupational stressors including lack of sleep [53], environmental stressors [54], psychogenic stress [55–57], or excess body fat [58]. Stress may be multifactorial in nature (i.e., combining environmental, emotional/cognitive, and metabolic stressors). *Menstrual dysfunction* may occur concomitant with, or separately from, menstrual disturbances and encompass abnormal physiological conditions associated with the menstrual cycle and include AUB such as heavy or prolonged bleeding [59], intermenstrual or chronic abnormal uterine bleeding [60] and/or painful bleeding (dysmenorrhea) [50]. Medical pathologies such as endometriosis [61] and polycystic ovarian syndrome (PCOS) [62] may be associated with both menstrual disturbances and menstrual dysfunction but are not within scope of this paper. Both menstrual disturbances and menstrual dysfunction may be influenced by a multistressor environment, but they may also contribute additional burden to a multistressor environment [63], thus highlighting the importance of management of menstrual disturbances and menstrual dysfunction of Servicewomen in operational situations. Table 2 defines and describes terms related to menstrual disturbances as further discussion of menstrual dysfunction goes beyond the scope of this paper.

**Table 2:** Terminology, hormonal profile, and presentation of menstrual disturbances

	Hormonal profile (sex steroids)	Presentation
**Primary amenorrhea**	Similar to secondary amenorrhea (also termed functional hypothalamic amenorrhea): low concentrations of E2 and P4 and lack of mid-cycle LH surge (anovulation) [65].	Delayed onset of menarche (≥ 15 years) and absence of development of secondary sexual characteristics, or menarche ≥ 16 years in presence of secondary sexual characteristics [66]. May be related to anomalies of the uterus, vagina, hymen, ovaries, or chromosomes as well as the HPO-axis [65].
**Secondary amenorrhea**	Similar to primary amenorrhea: low concentrations of E2 and P4 and lack of mid-cycle LH surge (anovulation), may be accompanied by overactive HPA and disturbance in HPT axes [67]. *Can only be diagnosed when other etiologies of amenorrhea have been excluded [67]	No menstrual bleeding for three consecutive cycles or longer (e.g., 90 days), and a negative LH surge test indicating anovulation [67]. May occur due to multistress environment but can also be hyperandrogenic in origin [68].
**Abnormal uterine bleeding infrequent (AUB-infrequent)** *Oligomenorrhea*	An erratic ovarian hormone profile, which may include evidence of **AUB-E**/*luteal phase defect* [69]. A positive mid-cycle LH surge (indicating ovulation may occur) is possible, but hormonal concentrations in the luteal phase likely do not support fertility [70, 71]. A negative LH surge test indicating anovulation and infertility is also possible [70, 71]	A menstrual cycle length of > 35–38 days [60]. May occur due to multistress environment but can also be hyperandrogenic in origin [68].
**Abnormal uterine bleeding – ovulatory dysfunction (AUB-O)** *Anovulation (without amenorrhea)*	Lack of mid-cycle LH surge (indicating ovulation) [51]. May be accompanied by suppressed concentrations of E2 and P4 throughout cycle [72]. May occur in the absence of amenorrhea.	Indicated by a negative LH surge test suggesting anovulation thus affecting fertility [51, 72]. May occur sporadically in more than a third of otherwise clinically normal cycles [73] and but may also become a chronic ovulatory disorder as in secondary amenorrhea of hypothalamic origin [51]. May also occur concurrently with **AUB-infrequent ** [72].
**Abnormal uterine bleeding – endometrial (AUB-E)** *Luteal phase defect (LPD)*	Suppressed P4 concentrations (< 16.0 nmol/L [10] or < 12.5 nmol/L [74]) during the luteal phase approx. 7 days post mid-cycle LH surge (indicating ovulation), although there is not agreement on a specific threshold for adequate progesterone or criteria for diagnosing LPD [75].	May be accompanied by short luteal phase (< 9 days) [76]. Associated with spotting (light bleeding before menstrual bleeding) [77], although spotting is not considered a definitive sign of **AUB-E** [75]. Associated with decreased fertility [78, 79] but has not been clearly identified as an independent entity affecting fertility [80]
**Heavy menstrual bleeding (HMB)** *Menorrhagia*	Several etiologies: • Endocrine dysfunction that interrupts the sequential events of the menstrual cycle may result in HMB [59, 81]. • Subclinical menstrual disturbance (including **AUB-E** and anovulation) may alter bleeding patterns (HMB or prolonged bleeding). This may be a result of E2 excess accompanied by low P4 (as P4 opposes biologic activity of E2 in the endometrium) [81]. • Low energy availability and accompanying subclinical menstrual disturbances may affect endometrial quality and thickness as well as reproductive hormonal milieu and iron status leading to HMB [82]. • For more etiologies see [64].	Abnormal uterine bleeding that is heavy (patient determined) or prolonged (> 7 days) menstrual bleeding that negatively affects daily function [64].

Terms in bold reflect updated terminology for sports and exercise science and medicine that are already used internationally in obstetrics and gynaecology [60] and described also in [51, 59, 64]. Older and possibly more familiar terminology is in italics

*AUB* abnormal uterine bleeding, *AUB-E* abnormal uterine bleeding – endometrial, *AUB-O* abnormal uterine bleeding – ovulatory, *E2* estradiol, *HMB* heavy menstrual bleeding, *HPA* hypothalamic-pituitary-adrenal, *HPT* hypothalamic-pituitary-thyroid, *LH* luteinizing hormone, *LPD* luteal phase defect, *P4* progesterone

### Early and Late Signs of Menstrual Disturbance

**Fig. 3:**
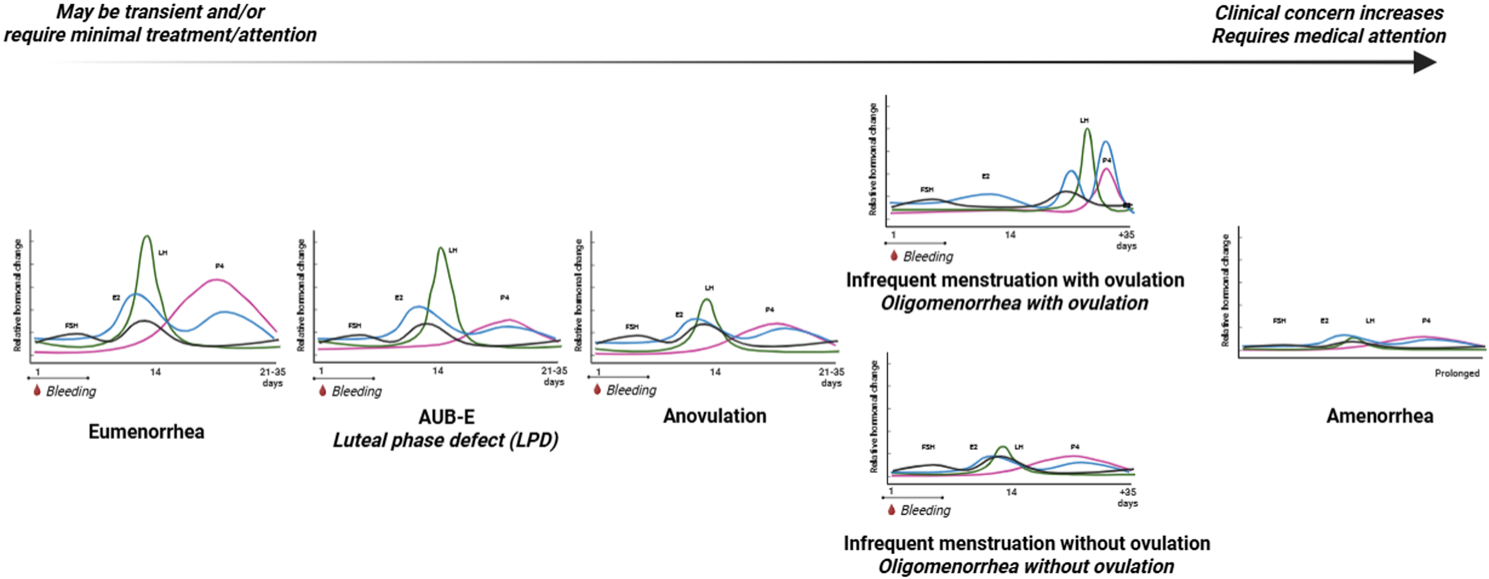
Schematic representation of menstrual disturbances: eumenorrhea, abnormal uterine bleeding–endometrial (AUB-E), anovulation, abnormal uterine bleeding-infrequent (AUB-infrequent), and amenorrhea. Figure created in Biorender

Endocrine responses to stressors can be considered adaptive or maladaptive (e.g., when menstrual disturbances occur). Menstrual disturbances occur on a wide spectrum ranging from subtle/subclinical to severe/clinical [83] (Fig. 3). A hallmark symptom of menstrual disturbance is AUB including a decrease in or lack of bleeding. For example, a prolonged menstrual cycle (AUB-infrequent), repeated cycles with anovulation (AUB-ovulatory dysfunction), a suppressed luteal phase (AUB-endometrial), or the complete absence of menstrual bleeding (amenorrhea), all indicate downregulation of the HPO axis (i.e., suppression of LH and FSH as well as decreased concentrations of P4 and E2) [60, 84, 85]. The presence of menstrual bleeding, however, is not confirmation of an ovulatory or “normal” eumenorrheic cycle or an “adequate” luteal phase [10] meaning that subtle/subclinical menstrual disturbances may go unnoticed.

Menstrual disturbances can result from low energy availability—as described in the Female Athlete Triad (Triad) [69, 86–90] and Relative Energy Deficiency in Sport (REDs) [52, 91, 92] frameworks—as a result of partitioning metabolic fuels for processes essential for survival (i.e., circulation and neural activity) from non-essential processes (i.e., reproduction) [93]. Menstrual disturbances can also be caused by other stressors including lack of sleep [53], psychological load [55, 56], environment [54], and / or excess body fat [58]. Finally, menstrual disturbances can be caused by gynecological conditions including PCOS or primary ovarian insufficiency (POI) [67]. Therefore, the endocrine system is sensitive and reactive to stressors encountered by women in the military [94]. While downregulation of the HPO axis can be functional in nature and serve to limit chances of reproductive success during times of energetic or psychological stress [95] prolonged suppression can be deleterious to overall health and performance [6].

### Heavy Menstrual Bleeding

When the sequential events of the menstrual cycle are disrupted, heavy menstrual bleeding (HMB) may be observed [59, 81]. Subclinical menstrual disturbances such as AUB-E and anovulation are associated with altered bleeding patterns causing HMB, intermenstrual bleeding (*metrorrhagia*) or chronic AUB. Endometrial quality and thickness can be affected by low energy availability and accompanying reproductive hormonal milieu, as well as iron status [82]. Heavy menstrual bleeding may also be caused by excess E2 and low P4, noting that P4 opposes biologic activity of E2 in the endometrium [81] and can increase burden and psychological stress as it requires additional considerations regarding hygiene. For specific terminology/classification of HMB see [51, 59, 60, 64].

### Menstrual Suppression with Hormonal Contraceptives

Hormonal contraceptive use is common in Servicewomen [96–98]. Hormonal contraceptives either partially or completely suppress HPO axis function depending on the exogenous hormone or hormones used, their dose, timing of use, and route of administration. A significant proportion of Servicewomen indicate they would like to manage and/or suppress menstrual cycles, especially when deployed [99]. In addition to family planning, menstrual suppression may be motivated by logistical challenges, to control bleeding, and to avoid the need for sanitary products and access to facilities to change them [8, 100]. While it is important to recognize that there is very limited research investigating the concomitant effects of menstrual suppression and multistressor environments on the (long-term) health and performance of Servicewomen, a thorough discussion of hormonal contraceptives extends beyond the scope of this paper. At present, the limited data show that hormonal contraceptive use does not markedly affect health or performance outcomes in response to military training but may impact bone [101].

### Prevalence of Menstrual Disturbances

Several studies have investigated menstrual disturbances in the military [18, 94, 102–109], but there are inconsistencies with definitions and screening methods used [57]. Cross-sectional data in trained US Army personnel from the 1990s identified prevalences of 12% for AUB-infrequent and amenorrhea (< 9 cycles in 12 months or 3 months with no cycle); 13% for primary amenorrhea (≥ 15 years) [102]; 15% of ever having amenorrhea (> 6 months without menses); and 4% with primary amenorrhea (≥ 17 years) [103]. In a large cohort of trained UK Servicewomen, the prevalences were 18% for AUB-infrequent or amenorrhea; 25% for history of amenorrhea; and 14% for primary amenorrhea citing risk factors for menstrual disturbances as younger age, poorer sleep, more field exercise, risk of eating disorders, and anxiety and depression [106]. The prevalence of menstrual disturbances appears higher in basic military training than in trained Servicewomen, with more than 65% of women undergoing basic military training reporting menstrual cycle disturbances or changes [18, 57, 104, 107–110]. At the US military academy, more than 90% of women reported menstrual disturbances during one year of basic military training with almost half of the female cohort reporting decreased menstrual frequency [18, 104]. Additional data from the US Army show that 86% of women experienced changes in their menstrual cycle during basic military training, and 41% missed one period [108]. Most women (85%) also reported a cessation of menses during US Marine Corps training [109]. A similarly high prevalence (70%) of menstrual disturbances was reported after starting Korean basic military training [107]. Data from British Army Officer basic training reported a prevalence of up to 65% of AUB-infrequent or amenorrhea, with 95% of women experiencing AUB-O [57]. This study also showed impaired responsiveness of the HPO axis after 28 weeks of training using a low-dose infusion of GnRH. Basic military training is one the most physically and psychologically arduous periods of a military career [4] and likely explains the higher prevalence of menstrual disturbances in trainees than in trained Servicewomen. These studies were observational and largely relied on recall for previous menstrual function, and so it is not clear which element of military service caused the change in menstrual function. Despite evidence of a high prevalence of menstrual disturbances in basic training, there is no evidence that women perform poorer because of these disturbances.

The prevalence of secondary amenorrhea (2% to 5%) [91, 111–113], AUB-infrequent/amenorrhea (11% to 14%) [113, 114], and primary amenorrhea (≥ 15 years) (10%) [115] appears higher in Servicewomen than the general population. The prevalence of menstrual disturbances is also higher in athletes than in the general population but varies widely by athlete group [111]; between 21% and 43% of recreational and elite endurance athletes report AUB-infrequent or amenorrhea [116–121], but the prevalence can be higher (> 60%) in sports that are aesthetic or dependent on being ‘lean’, and long-distance running [91, 111]. Data from athletes are typically based on young or adolescent women, whereas military studies often include women across reproductive ages and later gynecological age is associated with a lower prevalence of menstrual disturbances [106, 111]. Data from Servicewomen separated by age show the prevalence of AUB-infrequent or amenorrhea in young Servicewomen (17 to 24 years) was 32% [106], which is similar to athlete populations. However, inconsistencies with defining and measuring menstrual disturbances means comparisons between studies and populations should be made with caution.

## Military Stressors Affecting Menstrual Health

Military employment requires all soldiers to be ‘fit to fight’, and soldiers often train in austere conditions to simulate operations. Training and operational environments are typically characterized by high physical demands, constrained dietary choices and opportunities to eat, sleep deprivation/disruption, psychological and social stress (from both work and from being away from home and family), remote and exposed living conditions, and temperature extremes [122, 123]. These stressors independently or combined can contribute to menstrual cycle disturbances in a military environment (described below), but a general limitation of military field research is the inability to determine the independent effects of each stressor. Core military tasks are often physically arduous and involve heavy load carriage, manual material handling, and casualty evacuation that require adequate aerobic fitness, muscle strength, and muscle endurance [46]. These physical demands are absolute, requiring women to work relatively harder than their male counterparts [3, 4], and sudden and sustained increased total energy expenditures can lead to severe energy deficit, more so on field exercise [124, 125]. While recent data suggest men are at greater risk of energy deficit than women [124, 126], women may be more susceptible to downregulation of reproductive function than men in military training [127]. These operating environments can threaten menstrual and physical health, challenge the remote management of uterine bleeding, and influence hormonal contraceptive choices. Despite these additional challenges for Servicewomen, many women still perform well in some of the most demanding military roles.

### Energy Balance and Energy Availability

The high total energy expenditures in military training and operations [3, 4] means Servicewomen (and men) may be at risk of under fueling. The failure to meet required energy and nutrient needs may result in measurable changes in endocrine function, and cognitive and physical performance [3, 4], and if chronic, may be associated with micronutrient deficiencies [128]. Energy balance describes the difference between energy intake and total energy expenditure. Negative energy balance occurs when energy total expenditure exceeds energy intake [129]. Energy availability is the dietary energy available for metabolic function after exercise, defined as energy intake minus exercise energy expenditure expressed relative to fat free mass [129]. The measurement of energy availability in a military environment is challenging, thus most studies measure energy balance and indicate negative energy balances in many military training environments [3, 4, 130]. The innate smaller body size of women compared with men means women often better match their energy intake to their energy expenditure, which could help better protect lean mass [124, 126, 131]. Normalizing total energy expenditure and energy balance outcomes to body mass removes any sex-based differences [132].

Low energy availability results in suboptimal energy availability for other physiological functions in the body, such as maintenance of reproductive function [133]. The evidence linking low energy availability to menstrual disturbances are described in detail elsewhere [52, 69, 86–92]. A threshold for “optimal” energy availability defined as 45 kcal∙kg^− 1^ fat free mass∙day^− 1^, has been suggested to provide enough energy to maintain body mass, muscle hypertrophy, and support overall health, including menstrual function [134, 135]. The validity of this threshold in a military context and under operational multistressors has not yet been established. While short-term low energy availability can be accompanied by beneficial effects on health and performance (adaptable), prolonged or severe low energy availability can be accompanied by negative effects on some health and performance outcomes [6, 34, 52]. The evidence linking low energy availability to wider health and physical performance outcomes has been described in the Triad [69, 86–90] and REDs [52, 91, 92] frameworks. Military exposure to low energy availability may most commonly be through repeated exposures to acute bouts of low energy availability, the long-term impact of which is not clear [3, 4]. Low energy availability in exercising women appears to decrease insulin, E2, P4, leptin, and IGF-I, and increase ghrelin, peptide YY, insulin, cortisol, and growth hormone [135]. Similar effects are seen in men during military training in energy deficit, but there are comparatively less data in women [3, 4]. Menstrual disturbances induced by low energy availability may impair physical performance, where natural/eumenorrheic menstrual cycles might facilitate better adaptations to training and physical performance [5, 6] (as described below in Menstrual Disturbances and Occupational Performance). A more holistic understanding of the interaction between low energy availability, endocrine function, menstrual disturbances, and military relevant outcomes are required.

### Micronutrients

The most notable micronutrient deficiency associated with low energy availability is iron deficiency, where symptoms of iron deficiency and low energy availability can be similar. Iron deficiency can be a cause and result of HMB, although secondary amenorrhea may reduce iron requirements. Iron deficiency can compound low energy availability by shifting energy production to less efficient anaerobic pathways thus straining metabolic output [136]. Women have a 50% increased requirement for iron compared with men due to the natural loss of iron that occurs with menstruation [128]. Basic military training worsens iron status in women [137] with the decrements higher in women than men [138]. Poorer iron status has been associated with poor aerobic performance in female recruits [138]. Eight weeks of iron supplementation in young female recruits with iron-deficiency anemia in US Army basic training improved aerobic performance and mood [139]. Beyond performance, iron deficiency may contribute to poor bone health through suppression of GH and IGF-I [136, 140] as well as altered bone metabolism [138] and microstructure [141]. Studies have linked iron status to bone stress injury in Servicewomen; female recruits in the Israeli Defense Force (IDF) suffering from bone stress injury had reduced biomarkers of iron status [142]. In addition to iron supplementation, bone health of Servicewomen may be supported with adequate calcium and vitamin D intake; US Army female recruits receiving daily calcium and vitamin D supplementation during their eight-weeks of training showed improved bone mineral density outcomes [143]. Whilst there is no evidence for a link between vitamin D and calcium status and menstrual cycle status, strategies to protect bone health are essential in women with menstrual cycle disturbances.

### Microbiome

The gut microbiome acts to process dietary fibers, aid in digestion/absorption, and regulate immune function and overall health by protecting against pathogens [144]. In general, women have lower *Bacteroides* abundance but a higher microbial diversity than men [145, 146]. Recent work has examined sex-based differences and the contribution of the gut microbiome and diet to overall female health [147]. There appears a bidirectional relationship between the gut and estrogens as well as a functional role for the gut-estrogen relationship in female physiology and health [148]. Gut microbiome has been shown to affect systemic estrogens concentrations [149] and gut microbial diversity positively correlated with metabolites of estrogens [150]. The microbiome impacts secretion of the enzyme β-glucuronidase, deconjugates estrogens into their active forms, and suppresses circulating estrogens [151]. To date, little is known about the relationship between gut microbiota and menstrual cycle or hormonal contraceptive use, however, data show the presence and ratio of microbial species is altered in PCOS, cancer, pregnancy, and menopause [145]. Microbiome modulation could be a new tool for defense stakeholders [152] perhaps also to support menstrual health, but further research is necessary.

### Sleep

Restricted sleep is common in the military; average sleep duration during British Army infantry and non-infantry basic training was below 6 h per night [153], falling below recommendations of 7 to 9 h [154]. Similarly, British Army Officer Cadets who underwent 44 weeks of arduous training—and have a high incidence of menstrual disturbances—slept less than 6 h per night throughout training [57, 155]. Sleep disturbances suppress the HPO axis; specifically, poor sleep (quality, efficiency, and duration) has been associated with PMS, painful menstruation (dysmenorrhoea), and menstrual disturbances [156]. Women who slept less than 5–6 h compared with 8–9 h were more likely to experience menstrual disturbances [156]. This association between sleep and menstrual disturbances is consistent with data on Servicewomen, whereby 25% in the UK Armed Forces reported a history of amenorrhoea and 56% experienced HMB at some point during their service career; sleeping < 6 h compared with > 8 h doubled the risk of menstrual disturbances. The mechanism between sleep and menstrual disturbances is not clear but likely involves activation of the HPA axis and disruption of circadian rhythmicity [156].

### Psychological Stress

Women undertaking basic military training exhibit HPA activation alongside menstrual disturbances [57, 110]. Activation of the HPA axis is determined from cortisol, the primary biological marker of stress. Increased cortisol in basic military training courses [44, 110] is likely elicited by increased psychosocial stress [127, 157] but a healthy adaptation to stress, measured by the acute cortisol response to exogenous adrenocorticotrophin [44], have also been observed. The psychological effects on female combatants operating in austere and almost exclusively male environments are unknown. More women (22%) than men (16%) exposed to combat report symptoms of common mental health disorders [158] while common mental health disorders—specifically anxiety and depression—double the risk of menstrual disturbances [106], supporting psychogenic mechanisms of HPO suppression. The prevalence and mechanisms of HPO suppression may be confounded by high uptake of hormonal contraception [98]. The impact of metabolic and psychosocial stress on reproductive function in female combatants is not fully understood, but studies on primates demonstrate these stressors are likely to be additive [159]. The long-term consequences of psychological stress on women’s health and performance *through one’s career* warrant further investigation.

### Environmental Stressors

Servicewomen must operate and maintain performance across environments. Environmental stress may impact menstrual function, but menstrual disturbances may also influence physiological responses to environmental stress [54]. Both E2 and P4 have important mechanistic implications for ventilation and thermoregulation [160–164] but the clinical implications of hormonal fluctuation and menstrual disturbances on environmental injury risk are unclear.

In the context of heat stress, E2 is a potent vasodilator [165] and P4 is thermogenic [166, 167]. The increased core temperature observed in the luteal phase of the menstrual cycle [166, 168] could act as a primary driver for women’s potential increased risk for exertional heat illness [169, 170] but no empirical evidence exists to support this theory [171, 172]. While P4 is thermogenic, there is currently no evidence to suggest P4 influences risk of developing exertional heat illnesses. Hormonal contraceptives influence thermoregulation where progestin-only oral hormonal contraceptives increase the threshold for sweating and combined oral hormonal contraceptives (progestin combined with estrogen) do not [173]. While the differences in thermoregulation between men and women are relatively small during typical training in heat stress [174, 175] the effect of menstrual disturbances on thermoregulatory responses, and ultimately the risk of heat illness and adaptive capacity to heat, is understudied. The relationship between gut permeability and estrogens may be important during heat stress as increased gut permeability can elicit downstream inflammation associated with exertional heat stroke [176] but research within this context is lacking.

The role of E2 and P4 in thermoregulation has not been well studied under cold stress [177]. Where E2 enhances cutaneous vasodilation—potentially alleviating decrements in dexterity—[167, 168], P4 likely shifts the onset of shivering to a higher body temperature [178, 179]; but these shifts in thermoeffector function are inconsistent [180]. There is currently no evidence that E2 or P4 would be protective against cold-related injuries. More research is needed in this area to fully elucidate the potential impact of female sex hormones on thermoregulation. Furthermore, to our knowledge, there is currently no research evaluating the impact of suppressed hormonal function associated with menstrual disturbances on responses to cold stress.

Progesterone increases ventilation, which is important during high altitude or hypoxic exposures—both for performance and preventing acute mountain sickness—but its relative influence when evaluated in conjunction with E2, either via exogenous supplementation or endogenous production across menstrual cycle phase, remains unclear largely due to methodological differences between studies [181–183]. It has been hypothesized that P4 may alleviate onset of symptoms associated with acute mountain sickness by increasing ventilation, but recent findings have shown no influence of menstrual cycle phase on acute mountain sickness development [181]. In contrast, synthetic E2 and P4 included in combined oral contraceptives have been previously shown to increase the relative risk for developing acute mountain sickness in a sole investigation [184]. These findings are striking, indicating that lower endogenous P4 concentrations in oral contraceptive users may have influenced the increased prevalence of acute mountain sickness, but more evidence is needed. In hypoxic environments, which are encountered at high terrestrial elevations, the few investigations that include women report conflicting findings, specifically associated with ventilation responses (either increased or no effect) and effects on performance (either decreased or no effect) [163, 185–188].

High-altitude environments often coincide with cold stress. While there is limited information on cross-adaptation or the additive nature of these environmental stressors, it is possible that E2 and P4 play an important role in maintaining physiological function in terms of temperature regulation, dexterity, and ventilation. Additionally, menstrual disturbances may influence responses to environmental stress. Specifically, HMB may inhibit normal physiological responses in heat, cold, and high-altitude/hypoxia by reducing blood volume and potentially decreasing iron stores [189, 190]. More evidence is required to fully understand the influence of reproductive function and female sex hormones on the physiological responses to environmental extremes.

## Menstrual Disturbances and Occupational Performance

### Musculoskeletal Function and Injuries

Servicewomen are at an increased risk of musculoskeletal injuries compared with Servicemen [2], particularly bone stress injuries [191]. Estrogens play an important role in the development and maintenance of bone, mediating its actions on bone cells via the estrogen receptor alpha [192]. Estrogens protect bone by suppressing remodeling and may also increase the sensitivity of bone to mechanical loading [192]. The low E2 from secondary amenorrhea can harm musculoskeletal health [111, 193]. Therefore, changes to E2 status (e.g., menstrual disturbances [4] and hormonal contraceptive use [194–196]) may influence bone health in Servicewomen. Estrogens act on muscle, tendon, and ligament function, which may impact musculoskeletal injury risk [197]. In addition, estrogens may increase muscle performance by promoting sensitivity of muscle to anabolic signals [197] and acting as a neurosteroid in promoting neuromuscular performance [198].

Data from athletes show that those with menstrual disturbances (AUB-infrequent or amenorrhea) are at increased risk of bone stress [121, 199–201] and musculoskeletal [200, 202] injuries compared with their eumenorrheic counterparts. Menstrual disturbances can be accompanied by endocrine disturbances that can affect musculoskeletal tissues and can increase musculoskeletal injury risk; this endocrine disturbance can be as the result of the menstrual disturbance and/or from the independent effect of the stressor causing the menstrual disturbance (e.g., low energy availability) [4, 203]. There is some evidence from Servicewomen that menstrual disturbances are associated with increased risk of bone stress [103, 204–207] and musculoskeletal [208] injuries, however other data from Servicewomen show no association between menstrual status and bone mass [102, 209–211] or bone stress or musculoskeletal injury risk [203, 204, 210–214]. The lack of consistent pattern between menstrual status and musculoskeletal function and injuries in military studies could be due to differences in: (i) defining menstrual disturbances; (ii) methodologies used between studies; (iii) activities or training performed by Servicewomen (e.g., recruits in training *versus* trained personnel); (iv) location and type of the injury, and; (v) the relative influence of other risk factors (e.g., aerobic fitness). Also, military data may represent a ‘survivor’ cohort as a menstrual disturbance may increase the risk of leaving the military due to comorbidities or because of the stressors causing that menstrual disturbance [106]. A large study in British Servicewomen found that risk of an eating disorder or low energy availability (LEAF-Q score) were associated with increased prevalence of bone stress or musculoskeletal injuries, but having a menstrual disturbance was not associated with injury prevalence [203]. Low energy availability can directly disturb bone metabolism [215] and bone morphology [216] independent of menstrual function. Therefore, menstrual disturbances in athletes might increase risk of injury due to the non-reproductive, endocrine effects of low energy availability (e.g., decreased IGF-I), whereas menstrual disturbances in Servicewomen can be caused by stressors other than low energy availability, including poor sleep and psychological stress [4]; however, low energy availability may be caused by and the result of psychological stress.

### Physical Performance

Based on limited available data (e.g., specific to athletic populations with comprehensive menstrual evaluation included), meta-analyses indicate that the influence of hormonal fluctuations during a eumenorrheic menstrual cycle [29] and with hormonal contraceptive use [217] on physical performance (strength and / or endurance) is “trivial”, although individual experiences may differ and reports in the literature are conflicting. For example, fluctuations of E2 and P4 have been suggested to improve performance during the early follicular [218], ovulatory [219], and mid-luteal [220] phases, while no changes in performance outcomes across phases have also been reported [221–223].

The downstream effects of the broader endocrine dysfunction associated with a menstrual disturbance could result in physical performance decrements although data are limited; women with amenorrhea may have low E2 and P4, which are important hormones in the regulation and function of several physiological systems contributing to health and athletic performance [6]. Despite a high prevalence of menstrual disturbances in Servicewomen, there are limited data exploring menstrual disturbances and physical performance in this population. Research in athletic populations indicates that participants with ovarian suppression such as amenorrhea and infrequent menstruation (oligomenorrhea) experience reduction or stagnation in performance and development when compared with those with natural/eumenorrheic menstrual cycles [224–230]. Data from British Army basic training show that aerobic endurance and muscle strength performance adaptations to training are not different between combined oral contraceptive users, progestin-only contraceptives users, and non-users, but muscle strength is lower (albeit modestly) in oral contraceptive users [101], which could be linked to ovarian suppression via pharmacological means. Adding to the complexity of evaluating performance outcomes is the effect of the stressor causing menstrual disturbances (e.g., low energy availability), which can also directly impair physical performance through under-fueling [52].

### Cognition

Estradiol and P4 are highly lipophilic and easily pass through the blood-brain barrier. Several regions in the brain such as the amygdala, hypothalamus, and hippocampus express high densities of both E2 and P4 receptors, indicating the presence of sex steroid specific pathways [231] that may be influenced by menstrual disturbances. These receptors that are not only found in brain areas associated with reproduction but also found in areas that are important for cognitive function and emotional processing [232]. The neurotransmitters serotonin, dopamine, ɣ-aminobutyric acid, and glutamate—which are synthesized in the central nervous system and gastrointestinal tract—may be influenced by female sex hormones [232]. These neurotransmitters regulate mood, appetite, sleep, memory, and learning. Estradiol is mostly associated with positive effects on mood and cognition, while P4 is more related to negative mood effects, although these effects are rather complex and have a high interindividual variability. There are no data exploring how menstrual disturbances impact cognitive performance in Servicewomen. Therefore, inferences must be made from data drawn from other populations and using other models including the eumenorrheic menstrual cycle and hormonal contraceptive use.

Research on the role of the menstrual cycle in cognitive performance can be subdivided in emotion-related (e.g., emotional facial recognition and emotion processing) and emotion-independent cognition (e.g., visuospatial tasks) as the evidence differs for the role of the menstrual cycle in each type of cognition [233]. Within emotion-dependent cognitive processes there are associations between performance and menstrual cycle phase, with the luteal phase associated with poorer performance [233]. In contrast, within the emotion-independent cognition research, the evidence is less consistent. Traditionally, research on the role of the menstrual cycle in emotion-independent cognition has focused on sexually dimorphic cognitive abilities/skills (e.g., men are expected to outperform women in visuospatial tasks, whereas women are expected to outperform men in verbal tasks). Therefore, to obtain further insights in the role of sex hormones in cognition and clarify possible sex-related differences in cognitive performance, the menstrual cycle has been a useful model [233]. Although evidence suggests associations between emotion-independent cognitive processes and menstrual cycle phases, the evidence is largely inconclusive with insufficient evidence to support the hypothesis that cognitive tasks that men perform better than women would be improved in the follicular phase (i.e., the phase in which E2 and P4 are low) [234]. Relying on the few well-designed and powered research studies, there does not appear to be an effect of menstrual cycle phase on cognitive performance in both traditionally sex dimorphic tasks (e.g., visuospatial tasks, etc.) and executive functioning tasks [232, 233]. Further research is warranted, as transient fluxes in the menstrual cycle and inconsistent research methodologies have been proposed to play a significant role in the occurrence of inconsistent findings [233], while evaluation of menstrual disturbances and cognition are limited. Presently, however, data on the impact of PMS and PMDD in the general population suggest that there may be lower performance in some aspects of executive functioning in those who are most symptomatic [233]. In addition, Baskaran et al. (2017) observed that estrogen replacement improved performance on an immediate recall task and on an inhibition-switching task in young oligo-amenorrheic athletes with estrogen deficiency who underwent 6 months of estrogen replacement [235]. These results support the role of sex steroids in the regulation of higher cognitive functions.

There are no data on the cognitive effects of hormonal contraceptive use in Servicewomen and few data in athletes [236]. Oxfeldt et al. (2020) assessed the self-perceived physical and emotional symptoms related to the menstrual cycle/hormonal contraceptive cycle in elite female athletes [237]. This study found that hormonal contraceptive use was associated with reporting fewer positive and negative emotional symptoms, which suggests that hormonal contraceptive use might be a strategy to reduce emotional distress [237]. Evaluation of cognitive performance over the menstrual cycle has typically been performed with participants who have refrained from prolonged cognitive activity prior to testing rather than in a fatigued and / or sleep deprived state, which calls into question the applicability of these results in a multistressor environment.

## Lived Experiences of the Workforce

The impact of menstruation and menstrual disturbances on military service has not been well-established. The inter-relationship is complex given the mix of personal, logistical, and societal influences, and the fact that military employment may influence the menstrual cycle [94]. The experience of menstruation is a combination of physical symptoms, having appropriate sanitary products and locations for changing and disposal, and maintaining hygiene [100], all in the context of societal and organizational attitudes that tend to encourage concealment of these menstrual challenges. Female trainees who report changes in their menstrual cycle indicated that menstrual and premenstrual symptoms impacted academic, physical, and military activities, while reporting difficulties in obtaining/changing/disposing of menstrual materials in a military setting [18]. The considerations associated with menstruation also include medical conditions, which military medical systems must manage at home and on operations and deployments. There are important associations with menstrual disturbances, hormonal contraception, pregnancy, and family planning in the military career context. In aggregate, the experience of menstruation can influence Servicewomen’s overall military lived experience [100] and may be reflected in retention and occupational performance.

Retention is the continued employment of personnel with capabilities needed to conduct military operations. Retention is multifactorial, and several explanatory models exist; however, it can be summarized as a culmination of all personal and professional experiences and the many contributing factors. Retention is a strategic priority for many militaries because of the extensive and expensive training, education, and socialization needed to shape effective military members. A complicated mix of personal, organizational, and societal variables influence military attrition and retention, and it is dependent on factors such as job satisfaction, well-being, organizational commitment, and identity [238]. Isolating the role of menstruation, menstrual disturbances, and overall menstrual health over the life span, and its associated features on retention given the backdrop of other influences on Servicewomen and those who menstruate is challenging, but it is clearly essential to provide the support and conditions to thrive, including those associated with menstruation, to ensure a full and fulfilling career. Studies have shown that failure to provide the basics such as safe and supportive environments, properly fitting equipment and clothing, and adequate facilities for privacy and personal hygiene are a factor in retention [239].

## Call to Action

A strength of this review is the comprehensive overview of published evidence on menstrual disturbances in the military, but there are clear knowledge gaps that limit our conclusions. Therefore, we have drawn on evidence sources using different populations (e.g., athletes or recreationally active women) or different hormonal models (e.g., hormonal contraceptive use). Whilst our search was thorough, we did not use a systematic review methodology, and so it is possible that evidence sources were missed.

A collaborative effort across militaries is needed to focus research and funding on menstrual health in the military context, including exploring strategies to prevent menstrual disturbances/dysfunction; education on menstrual suppression (hormonal contraceptive use); and measures to restore menstrual function. Furthermore, a better understanding regarding the influence of sex hormone concentrations on performance and the effect of menstrual (or hormonal contraceptive) cycle symptoms on occupational performance is warranted. The physical requirements of military tasks and environments are unique, and Servicewomen face exceptional challenges. Despite these exceptional challenges, Servicewomen perform well in military roles, and better support to reduce some of these challenges will only enhance their performance and experience. As such, Servicewomen require specific research effort to meet their needs and to maximize their potential. Unlike athletes, Servicewomen have little to no control over their individual training schedules, volumes, and recovery periods. In addition, Servicewomen operate in multistressor environments where the relationship between menstrual health, overall health, and performance (physical and cognitive) are important to understand and support. Below is a brief list of gaps in knowledge requiring focused research efforts to support Servicewomen:

- Examine the relationship between menstrual disturbances/dysfunction and tactical, physical, and cognitive performance in an operational environment.
- Identify modifiable and non-traditional (e.g., psychogenic, microbiome) factors that influence menstrual health that could be targeted in support of Servicewomen.
- Explore the role of sex steroid hormones (endogenous and exogenous) on military relevant physical and cognitive performance in operationally relevant environments.

## Conclusions

Women in military employment are at a high risk of menstrual disturbances, particularly during arduous training and stressful operations where fuel and sleep may be limited and there is exposure to psychological stress and environmental extremes. Menstrual disturbances in Servicewomen, as in athletes, may increase the risk of other negative outcomes, including musculoskeletal injury and impaired physical and cognitive performance, although data are limited. The risk of menstrual disturbances in Servicewomen is likely exacerbated by their unique multistressor environments, warranting further research and resources specific to this population. The potential widespread impact of endocrine function on the health and performance of Servicewomen highlights the need for adequate resources, education, and healthcare to address overall menstrual health in the military.

## Data Availability

No data were used in the production of this manuscript.

## Abbreviations

ACTH: Adrenocorticotropic hormone
AUB: Abnormal uterine bleeding
AUB-E: Abnormal uterine bleeding – endometrial
AUB-O: Abnormal uterine bleeding – ovulatory
E2: 17β-estradiol
FSH: Follicle stimulating hormone
GH: Growth hormone
GnRH: Gonadotropin-releasing hormone
HMB: Heavy menstrual bleeding
HPA: Hypothalamic-pituitary-adrenal
HPO: Hypothalamic-pituitary-ovarian
HPT: Hypothalamic-pituitary-thyroid
IDF: Israeli Defense Force
IGF-I: Insulin-like growth factor-I
LH: Luteinizing hormone
LPD: Luteal phase defect
P4: Progesterone
PCOS: Polycystic ovarian syndrome
PMDD: Premenstrual dysphoric disorder
PMS: Premenstrual syndrome
POI: Primary ovarian insufficiency
REDs: Relative Energy Deficiency in Sport
T: Testosterone
Triad: Female Athlete Triad

## Declarations

The opinions or assertions contained herein are the private views of the authors and are not to be construed as official or as reflecting the views of the United States Army, British Army, Royal Military Academy of Belgium, or the Canadian Armed Forces.
